# Assessing mortality risk in Type 2 Diabetes patients with prolonged ASCVD risk factors: the inclusive Poh-Ai predictive scoring system with CAC Score integration

**DOI:** 10.1186/s13098-024-01341-9

**Published:** 2024-05-19

**Authors:** Meng-Huan Lei, Yu-Chen Hsu, Sheng-Liang Chung, Chao-Chin Chen, Wei-Cheng Chen, Wan-Ming Chen, An-Tzu Jao, Ju-Feng Hsiao, Jen-Te Hsu, Szu-Yuan Wu

**Affiliations:** 1 Division of Cardiology, Department of Internal Medicine, Lo-Hsu Medical Foundation, Lotung Poh-Ai Hospital, No. 83, Nanchang St., Luodong Township, Yilan County, 265 Taiwan; 2 Graduate Institute of Business Administration, College of Management, Fu Jen Catholic University, New Taipei City, Taiwan; 3 Artificial Intelligence Development Center, Fu Jen Catholic University, New Taipei City, Taiwan; 4 Big Data Center, Lo-Hsu Medical Foundation, Lotung Poh-Ai Hospital, No. 83, Nanchang St., Luodong Township, Yilan County, 265 Taiwan; 5 Department of Food Nutrition and Health Biotechnology, College of Medical and Health Science, Asia University, Taichung, Taiwan; 6 Division of Radiation Oncology, Lo-Hsu Medical Foundation, Lotung Poh-Ai Hospital, Yilan, Taiwan; 7 Department of Healthcare Administration, College of Medical and Health Science, Asia University, Taichung, Taiwan; 8 Centers for Regional Anesthesia and Pain Medicine, Taipei Municipal Wan Fang Hospital, Taipei Medical University, Taipei, Taiwan

**Keywords:** Type 2 diabetes, Atherosclerotic cardiovascular disease, All-cause mortality, Predictive scoring system, Coronary artery calcium

## Abstract

**Purpose:**

To enhance the predictive risk model for all-cause mortality in individuals with Type 2 Diabetes (T2DM) and prolonged Atherosclerotic Cardiovascular Disease (ASCVD) risk factors. Despite the utility of the Coronary Artery Calcium (CAC) score in assessing cardiovascular risk, its capacity to predict all-cause mortality remains limited.

**Methods:**

A retrospective cohort study included 1929 asymptomatic T2DM patients with ASCVD risk factors, aged 40–80. Variables encompassed demographic attributes, clinical parameters, CAC scores, comorbidities, and medication usage. Factors predicting all-cause mortality were selected to create a predictive scoring system. By using stepwise selection in a multivariate Cox proportional hazards model, we divided the patients into three risk groups.

**Results:**

In our analysis of all-cause mortality in T2DM patients with extended ASCVD risk factors over 5 years, we identified significant risk factors, their adjusted hazard ratios (aHR), and scores: e.g., CAC score > 1000 (aHR: 1.57, score: 2), CAC score 401–1000 (aHR: 2.05, score: 2), and more. These factors strongly predict all-cause mortality, with varying risk groups (e.g., very low-risk: 2.0%, very high-risk: 24.0%). Significant differences in 5-year overall survival rates were observed among these groups (log-rank test < 0.001).

**Conclusion:**

The Poh-Ai Predictive Scoring System excels in forecasting mortality and cardiovascular events in individuals with Type 2 Diabetes Mellitus and extended ASCVD risk factors.

**Supplementary Information:**

The online version contains supplementary material available at 10.1186/s13098-024-01341-9.

## Introduction

In asymptomatic patients, the presence of coronary artery calcification (CAC) serves as a well-established marker of subclinical atherosclerosis [1–3]. CAC imaging offers numerous advantages over procedures like coronary computed tomography angiography, such as requiring minimal patient preparation, obviating the need for iodinated intravenous contrast, and exposing patients to low effective radiation doses [2, 3]. Clinically, CAC plays a vital role in predicting atherosclerotic cardiovascular disease (ASCVD) risk and can be instrumental in guiding the prevention and treatment of ASCVD [4, 5], including recommending interventions like aspirin, statins, and advocating aggressive lifestyle modifications [6, 7].

The intricate interplay between Type 2 Diabetes (T2DM) and ASCVD significantly heightens the vulnerability to cardiovascular events and premature mortality [8, 9]. Early intervention and precise risk assessment are pivotal in the proficient management of this high-risk cohort. The introduction of predictive scores for all-cause mortality among asymptomatic individuals with concurrent T2DM and ASCVD risk factors extending over five years is undeniably indispensable [9–13]. Many of these individuals may remain oblivious to their heightened mortality risk [9–13]. Initiating an educational process and integrating a comprehensive risk assessment tool can expedite timely interventions. This may encompass optimizing diabetes control, instituting preventive strategies for heart disease, including aspirin or statin therapy, or implementing tailored lifestyle modifications, all aimed at the overarching objective of curbing all-cause mortality [14].

Despite the valuable role of the CAC score in evaluating coronary artery calcification and forecasting cardiac events [1–3], its aptness for predicting all-cause mortality remains suboptimal, despite the fact that cardiovascular disease (CVD) stands as a predominant contributor to mortality among individuals with T2DM, responsible for nearly half of all recorded fatalities [9]. The risk of mortality in individuals with T2DM and ASCVD risk factors over 5 years involves a multifaceted interplay of variables, including age, gender, comorbidities, diabetes duration, and medication use, among others [10]. The CAC score, proficient in assessing cardiovascular outcomes, primarily revolves around coronary artery calcification and may not encompass the broader spectrum of factors contributing to all-cause mortality. To address this limitation, we enhance the predictive risk model by building upon the well-established CAC scores for T2DM patients with ASCVD risk factors persisting over 5 years. This comprehensive model extends its purview beyond cardiovascular aspects, incorporating demographic and clinical parameters for a thorough assessment of mortality risk. Such predictive scores are pivotal in empowering healthcare professionals to promptly identify high-risk individuals and initiate precise interventions. By mitigating the constraints of the CAC score and emphasizing the prediction of all-cause mortality, these novel models hold promise for advancing risk management and, ultimately, ameliorating outcomes and the overall quality of life for individuals contending with the challenges of T2DM and ASCVD.

## Patients and methods

### Patients

In this retrospective cohort study, conducted with institutional review board approval from Lo-Tung Poh-Ai Hospital (review board number OMCP-97-013), we enrolled 1929 consecutive asymptomatic T2DM patients through the Lan-Yan Diabetes Shared Care Network spanning from August 2006 to August 2007. Inclusion criteria encompassed individuals aged 40–80 years with T2DM and at least 5 years of ASCVD risk factors, while exclusion criteria comprised documented coronary artery disease, typical angina, abnormal resting electrocardiogram findings (e.g., Q waves and left bundle branch block), cerebrovascular or peripheral arterial disease, or severe life-threatening illness. Written informed consent for baseline and follow-up data collection was obtained from all enrolled T2DM patients, who also underwent CAC score measurements, with the index date being defined as the date of CAC score assessment.

### Variables

Demographic attributes and clinical parameters were comprehensively evaluated, encompassing CAC scores, age, gender, body mass index (BMI), waistline measurements, cigarette smoking habits, and an array of laboratory data including total cholesterol, low-density lipoprotein (LDL), high-density lipoprotein (HDL), glycated hemoglobin (HbA1c), fasting blood glucose, postprandial blood glucose, blood urea nitrogen, creatinine, triglyceride levels, as well as markers for microalbuminuria, macroalbuminuria, and blood pressure. Additional variables encompassed the duration of diabetes, presence of hypertension, cardiovascular discomfort symptoms, peripheral arterial occlusive disease, a family history of coronary artery disease, physical examination findings such as body weight, height, hip circumference, and waist-to-hip ratio, along with assessments for abnormal cardiac rhythms, hypercholesterolemia, proteinuria, and the presence of exertional dyspnea, stable angina, or atypical chest pain. Medication use, specifically statins and anti-diabetes medications, was also documented (Table 1). Notably, it is essential to emphasize that the allocation of points and establishment of cut-off values for each variable within the scoring system were based on standard values derived from laboratory data at Poh-Ai Hospital.

**Table 1:** Assessing all-cause mortality risk in Type 2 diabetic patients with atherosclerotic cardiovascular disease risk factors of over 5 years' duration, undergoing cardiac calcium scoring

	No mortality		Mortality		P-value
	N = 1718		N = 211		
	N	%	N	%	
CAC score					< 0.0001
0	438	25.49%	26	12.32%	
1–100	727	42.32%	74	35.07%	
101–400	340	19.79%	52	24.64%	
401–1000	127	7.39%	34	16.11%	
> 1000	85	4.95%	25	11.85%	
Missing	1	0.06%	0	0.00%	
Age (mean ± SD), years-old	1718	63.66 ± 9.17	211	69.32 ± 7.73	< .0.0001
Age, median (IQR, Q1, Q3), years-old	1718	65.00 (57.00,70.00)	211	71.00 (65.00,75.00)	< 0.0001
Age group, years	1718		211		< 0.0001
≤ 50	161	9.37%	3	1.42%	
51–60	449	26.14%	28	13.27%	
61–70	686	39.93%	70	33.18%	
≥ 71	422	24.56%	110	52.13%	
Sex					0.0003
Female	912	53.08%	84	39.81%	
Male	806	46.92%	127	60.19%	
BMI, Kg/m^2^; Mean (SD)	1674	26.96 ± 4.89	204	25.72 ± 3.87	0.0005
Median (IQR)	1674	26.49 (24.25,29.02)	204	25.25 (22.88,28.08)	< 0.0001
BMI, Kg/m^2^					0.0021
< 18.5	370	21.54%	70	33.18%	
18.5 ≤ BMI < 24	561	32.65%	63	29.86%	
24 ≤ BMI < 27	691	40.22%	68	32.23%	
27 ≤ BMI < 35	52	3.03%	3	1.42%	
≧35	44	2.56%	7	3.32%	
Waistline, cm; Mean (SD)	755	88.62 ± 9.37	99	88.00 ± 8.62	0.5345
Median (IQR)	755	89.00 (82.00,94.00)	99	88.00 (82.00,93.00)	0.3916
Waistline, cm					0.1156
Normal	242	14.09%	41	19.43%	
High	513	29.86%	58	27.49%	
Missing	963	56.05%	112	53.08%	
Cigarette Smoking					0.0027
Never	1205	70.14%	130	61.61%	
Still smoking	302	17.58%	48	22.75%	
Quit	211	12.28%	32	15.17%	
Missing	0	0.00%	1	0.47%	
Laboratory data					
Total cholesterol, mean (SD)	1603	202.75 ± 41.92	191	200.22 ± 44.88	0.4340
Median (IQR)	1603	201.00 (173.00,225.10)	191	199.00 (166.00,228.00)	0.4313
Total cholesterol > 200 mg/dl	1718	914	53.20%	100	0.2601
LDL, mean (SD)	1205	126.97 ± 50.77	139	126.19 ± 36.62	0.8606
Median (IQR)	1205	123.00 (102.00,146.00)	139	125.00 (101.00,148.00)	0.8304
LDL > 130 mg/dl	466	27.12%	47	22.27%	0.3009
HDL, mean (SD)	1223	44.20 ± 13.50	143	39.71 ± 11.91	0.0001
Median (IQR)	1223	42.00 (35.00,51.00)	143	39.00 (32.00,45.00)	0.0001
HDL < 35 mg/dl	305	17.75%	48	22.75%	0.0034
Blood pressure					
SBP, mmHg, mean (SD)	1709	136.42 ± 16.24	209	137.61 ± 17.01	0.3218
Median (IQR)	1709	132.00 (128.00,146.00)	209	138.00 (130.00,150.00)	0.1926
SBP, mmHg	1718		211		0.0965
< 120	139	8.09%	19	9.00%	
120–139	871	50.70%	86	40.76%	
140–159	519	30.21%	77	36.49%	
≥ 160	180	10.48%	27	12.80%	
Missing	9	0.52%	2	0.95%	
DBP, mmHg, mean (SD)	1709	80.84 ± 20.24	209	79.11 ± 10.58	0.2225
Median (IQR)	1709	80.00 (70.00,90.00)	209	80.00 (70.00,88.00)	0.1839
DBP, mmHg	1718		211		0.7630
< 80	672	39.12%	89	42.18%	
80–89	589	34.28%	72	34.12%	
90–99	335	19.50%	36	17.06%	
≥ 100	113	6.58%	12	5.69%	
Missing	9	0.52%	2	0.95%	
HBA1C, mean (SD)	1300	7.98 ± 1.59	157	8.19 ± 1.89	0.1256
Median (IQR)	1300	7.70 (6.80,8.80)	157	7.80 (6.80,9.20)	0.3006
HBA1C	1718		211		0.4949
< 6.5	183	10.65%	22	10.43%	
6.5–8.4	703	40.92%	76	36.02%	
≥ 8.5	414	24.10%	59	27.96%	
Missing	418	24.33%	54	25.59%	
Fasting blood glucose, mean (SD)	1630	159.11 ± 52.51	196	166.48 ± 68.38	0.0732
Median (IQR)	1630	148.00 (124.00,179.00)	196	150.50 (121.00,196.50)	0.5820
Glucose AC	1718		211		0.0029
< 100	80	4.66%	20	9.48%	
100–250	1442	83.93%	157	74.41%	
> 250	108	6.29%	19	9.00%	
Unknown	88	5.12%	15	7.11%	
Glucose PC, mean (SD)	656	209.84 ± 78.62	92	235.93 ± 98.91	0.0041
Median (IQR)	656	199.50 (149.00,254.00)	92	213.50 (170.50,285.50)	0.0276
Blood urea nitrogen, mean (SD)	1005	17.59 ± 8.18	141	24.65 ± 18.16	< 0.0001
Median (IQR)	1005	16.00 (13.00,20.00)	141	19.00 (14.00,27.20)	< 0.0001
Creatinine, mean (SD)	1407	1.18 ± 5.71	178	1.51 ± 1.41	0.4349
Median (IQR)	1407	0.90 (0.80,1.10)	178	1.10 (0.90,1.60)	< 0.0001
Triglycerides, mean (SD)	1520	165.82 ± 118.04	178	185.35 ± 133.16	0.0396
Median (IQR)	1520	136.00 (95.50,198.50)	178	149.00 (103.00,214.00)	0.0383
Microalbuminuria (30–300 mg)	576	33.53%	82	38.86%	0.1229
Macroalbuminuria (> 300 mg)	218	12.69%	45	21.33%	0.0005
Diabetes duration, years	1702	6.76 ± 5.94	208	8.40 ± 6.24	0.0002
	1702	5.00 (2.00,10.00)	208	8.00 (3.00,10.00)	< 0.0001
Diabetes duration, years	1718		211		0.0146
< 11	1433	83.41%	159	75.36%	
≥ 11	269	15.66%	49	23.22%	
Missing	16	0.93%	3	1.42%	
Concurrent comorbidities and familial medical background					
Hypertension	1212	70.55%	157	74.41%	0.2437
Cardiovascular discomfort symptoms	676	39.35%	72	34.12%	0.1416
Peripheral arterial occlusive disease	14	0.81%	6	2.84%	0.0060
Family history of coronary artery disease	136	7.92%	4	1.90%	0.0015
Physical examination					
Body weight (Kg), mean (SD)	1677	67.81 ± 11.47	204	65.38 ± 11.66	0.0044
Median (IQR)	1677	67.00 (60.00,75.00)	204	65.00 (57.25,72.75)	0.0036
Height (cm), mean (SD)	1674	158.62 ± 8.40	204	159.29 ± 8.28	0.2871
Median (IQR)	1674	158.00 (153.00,165.00)	204	159.00 (153.00,165.00)	0.3315
Hip circumference (cm), mean (SD)	751	96.54 ± 7.81	99	94.54 ± 8.00	0.0171
Median (IQR)	751	96.00 (91.00,101.00)	99	93.00 (90.00,99.00)	0.0063
Waist-to-hip ratio, mean (SD)	751	0.92 ± 0.07	99	0.93 ± 0.07	0.0572
Median (IQR)	751	0.92 (0.87,0.96)	99	0.93 (0.89,0.98)	0.0518
Waist-to-hip ratio					0.4655
Normal	148	8.61%	16	7.58%	
High	603	35.10%	83	39.34%	
Unknown	967	56.29%	112	53.08%	
Symptoms and signs					
Abnormal cardiac rhythm	2	0.12%	0	0.00%	0.6200
Hypercholesterolemia	1320	76.83%	144	68.25%	0.0059
Proteinuria	696	40.51%	108	51.18%	0.0030
Exertional dyspnea	519	30.21%	70	33.18%	0.3774
Stable angina	69	4.02%	7	3.32%	0.6224
Atypical chest pain	446	25.96%	59	27.96%	0.5325
Medication use					
Statin use	529	30.79%	54	25.59%	0.2796
Anti-diabetes Medications	1718		211		0.0259
Oral Hypoglycemic Agents	1460	84.98%	170	80.57%	
Insulin	35	2.04%	11	5.21%	
Combination of Oral Hypoglycemic Agents and Insulin	73	4.25%	12	5.69%	
Unknown	150	8.73%	18	8.53%	
Fasting Blood Glucose, Mean (SD)	1630	159.11 ± 52.51	196	166.48 ± 68.38	0.0732
Median (IQR)	1630	148.00 (124.00,179.00)	196	150.50 (121.00,196.50)	0.5820
Glucose AC	1718		211		0.0029
< 100	80	4.66%	20	9.48%	
100–250	1442	83.93%	157	74.41%	
> 250	108	6.29%	19	9.00%	
Unknown	88	5.12%	15	7.11%	
Glucose PC, Mean (SD)	656	209.84 ± 78.62	92	235.93 ± 98.91	0.0041
Median (IQR)	656	199.50 (149.00,254.00)	92	213.50 (170.50,285.50)	0.0276
Blood urea nitrogen, Mean (SD)	1005	17.59 ± 8.18	141	24.65 ± 18.16	< 0.0001
Median (IQR)	1005	16.00 (13.00,20.00)	141	19.00 (14.00,27.20)	< 0.0001
Creatinine, Mean (SD)	1407	1.18 ± 5.71	178	1.51 ± 1.41	0.4349
Median (IQR)	1407	0.90 (0.80,1.10)	178	1.10 (0.90,1.60)	<0.0001
Triglycerides, Mean (SD)	1520	165.82 ± 118.04	178	185.35 ± 133.16	0.0396
Median (IQR)	1520	136.00 (95.50,198.50)	178	149.00 (103.00,214.00)	0.0383

*CAC*: Coronary Artery Calcium; *IQR*: Interquartile Range; *SD*: Standard Deviation; *m*^*2*^: Square Meters; *BMI*: Body Mass Index; *LDL*: Low-Density Lipoprotein; *HDL*: High-Density Lipoprotein; *SBP*: Systolic Blood Pressure; *DBP*: Diastolic Blood Pressure; *HBA1C*: Glycated Hemoglobin; *Glucose AC*: Fasting Blood Glucose after a meal; *Glucose PC*: Postprandial Blood Glucose; *Kg*: Kilograms; *cm*: Centimeters; *N*: Numbers

### Statistical analysis

We conducted all statistical analyses using SAS for Windows (version 9.4; SAS Institute, Cary, NC, USA), with statistical significance established at P < 0.05. Crucial demographic parameters, including gender and age, were stratified and categorized accordingly, with patient age determined at the index date. The primary variables of interest encompassed CAC scores, Age, Sex, BMI, Waistline, Cigarette Smoking, Laboratory data, diabetes duration, hypertension, cardiovascular discomfort symptoms, peripheral arterial occlusive disease, family history of coronary artery disease, physical examination findings, comorbidities, and medication usage. To assess differences between two groups, both chi-square tests and t-tests were employed. Specifically, t-tests were used for evaluating dependent quantitative variables in relation to independent categorical variables with two groups, while chi-square tests of independence were applied to examine the association between two categorical variables.

Significant factors were identified to construct the Poh-Ai Predictive Scoring System for all-cause mortality in T2DM. We used the forward stepwise selection method to choose factors significantly predicting all-cause mortality (P < 0.05; Table 2). Factors with a coefficient of > 0 or an adjusted hazard ratio (aHR) of > 1 were selected as risk factors for the predictive scoring system, with points added according to the aHR [15]. The Cox regression model was utilized for the derivation of the score. Points were assigned based on the values of aHR, rounded to the nearest integer, via stepwise multivariate Cox proportional hazards model for all-cause mortality in T2DM patients with ASCVD risk factors persisting for over 5 years. Nonsignificant factors were eliminated from the model using a modified forward selection technique in the stepwise method. Duplicate entry and removal approaches were employed for forward selection and backward elimination. The "minimum F-to-enter" criterion was applied. The model with the lowest Akaike information criterion (AIC) was selected for estimating T2DM mortality with ASCVD Risk Factors over 5 Years [16]. We categorized patients into three risk groups based on their risk scores, predicting higher mortality for those with moderate to high risk scores. Receiver operating characteristic (ROC) curves were created for each score, with areas under the ROC curves determined. The DeLong test was used to rigorously compare diagnostic test performance (Poh-Ai and CAC predictive scoring system) by assessing differences in their ROC curve areas. [17] Kaplan–Meier analysis evaluated all-cause mortality using the Poh-Ai and CAC predictive scoring system, and the log-rank test determined differences among risk groups. A two-tailed P value of < 0.05 indicated statistical significance.

**Table 2:** Stepwise multivariate cox proportional hazards model for all-cause mortality in Type 2 diabetic patients with atherosclerotic cardiovascular disease risk factors persisting for over 5 years

Factor	aHR*	95% CI		*P* value	Assigned points
CAC score > 1000	1.57	1.04	3.37	0.0433	2
CAC score 401–1000	2.05	1.05	4.01	0.0353	2
CAC score 101–400	1.56	1.16	2.84	0.0237	2
CAC score 1–100	1.11	1.02	1.99	0.0452	1
Current Smoking	1.79	1.25	2.54	0.0013	2
Macroalbuminuria	1.72	1.20	2.48	0.0033	2
Age ≥ 71 years-old	9.72	2.34	40.44	0.0018	10
Age 61–70 years-old	4.40	1.06	18.33	0.0421	4
Age 51–60 years-old	3.32	1.18	14.17	0.0158	3
Glucose AC ≥ 250	1.66	1.05	2.92	0.0362	2
Diabetes Duration ≥ 11 years	1.55	1.07	2.25	0.0210	2

*CAC*: Coronary Artery Calcium; *Glucose AC*: Fasting Blood Glucose after a meal; *aHR*: Adjusted Hazard Ratio; *HR*: Hazard Ratio; *CI*: Confidence Interval

*Adjustment for all covariates in Table 1 was performed

## Results

### Demographics of T2DM patients with ASCVD risk factors over 5 years

We compared demographic characteristics, CAC scores, age, sex, BMI, waistline, cigarette smoking, laboratory data, diabetes duration, hypertension, cardiovascular discomfort symptoms, peripheral arterial occlusive disease, family history of coronary artery disease, physical examination, comorbidities, and medication use between non-mortality and mortality groups of T2DM patients with ASCVD Risk Factors Over 5 Years. Out of the 1929 patients included, 211 (10.9%) experienced mortality, while 1718 did not. Mortality was associated with higher CAC scores, older age, more male patients, lower BMI, a higher prevalence of smoking history, current smoking habits, lower HDL levels, higher glucose AC and PC, elevated BUN, creatinine, and triglycerides, increased macroalbuminuria, longer Diabetes Duration, a higher proportion of patients with Diabetes Duration > 11 years, more Peripheral Arterial Occlusive Disease, an increased frequency of Family History of Coronary Artery Disease, lower body weight, reduced hip circumference, a higher prevalence of abnormal cardiac rhythm, more cases of hypercholesterolemia, increased insulin use, and a higher incidence of Combination of Oral Hypoglycemic Agents and Insulin, compared to T2DM patients who did not experience mortality (Table 1).

### Selecting mortality predictors in type 2 diabetes with ASCVD risk factors

Table 2 presents all significant factors derived from the stepwise method in the multivariate model for variable selection. Each risk factor was assigned a score based on its HR. Following the stepwise selection in the multivariate Cox proportional hazards model for all-cause mortality in T2DM Patients with ASCVD Risk Factors Over 5 Years, the following factors were identified as significant independent risk factors for all-cause mortality along with their respective adjusted hazard ratios (aHR) and assigned scores: CAC score > 1000 (aHR: 1.57, score: 2), CAC score 401-1000 (aHR: 2.05, score: 2), CAC score 101-400 (aHR: 1.56, score: 2), CAC score 1-100 (aHR: 1.11, score: 1), Current Smoking (aHR: 1.79, score: 2), macroalbuminuria (aHR: 1.72, score: 2), Age ≥ 71 years-old (aHR: 9.72, score: 10), Age 61-70 years-old (aHR: 4.40, score: 4), Age 51-60 years-old (aHR: 3.32, score: 3), Glucose AC ≥ 250 (aHR: 1.66, score: 2), and Diabetes Duration ≥ 11 years (aHR: 1.55, score: 2). These factors significantly contribute to the prediction of all-cause mortality.

### Assessing all-cause mortality with Poh-Ai scoring

Our results undeniably establish a strong correlation between the accumulation of risk scores and the associated mortality rates, as evident from the following percentages: The risk of all-cause mortality differs across various score ranges: [score: 0–5, 2.0%], [score: 6–7, 4.0%], [score: 8–9, 9.0%], [score: 10–13, 15.0%], and [score: 14–20, 24.0%]. These risk groups, meticulously classified to represent varying degrees of vulnerability, include the following: very low-risk (score 0–5), low risk (score 6–7), moderate-risk (score 8–9), high-risk (score 10–13), and very high-risk (score 14–20), as detailed in Table 4.

### Assessing all-cause mortality with CAC scoring

**Fig. 1:**
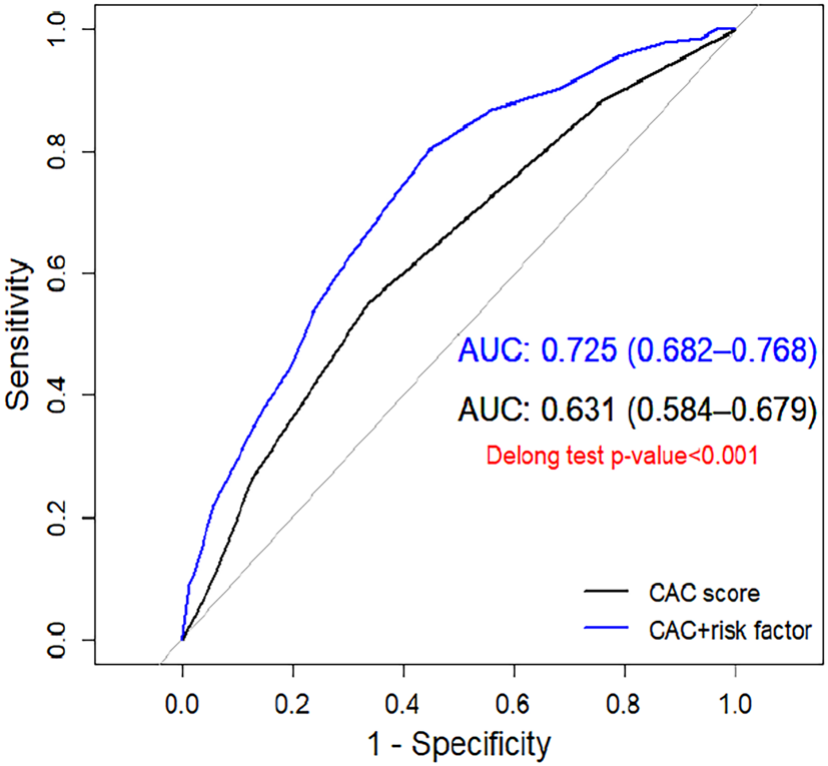
ROC Curves: Areas under the curve for Poh-Ai predictive and CAC scores in relation to all-cause mortality

The observed data showcases a distinct pattern in all-cause mortality rates: [0, 5.5%], [1–100, 8.6%], [101–400, 14.0%], [401–1000, 21.9%], and [> 1000, 17.0%] (Table 4). Furthermore, our meticulous analysis included the computation of areas under the ROC curves, with CAC scores achieving a value of 0.63 and Poh-Ai scores surpassing at 0.73 (as depicted in Fig. 1, Delong test < 0.001). This reinforces the superior predictive capacity of the Poh-Ai scoring system compared to CAC scores, highlighting its excellence in predicting all-cause mortality.

### Survival analysis: Poh-Ai and CAC predictive scoring systems for all-cause mortality

**Fig. 2:**
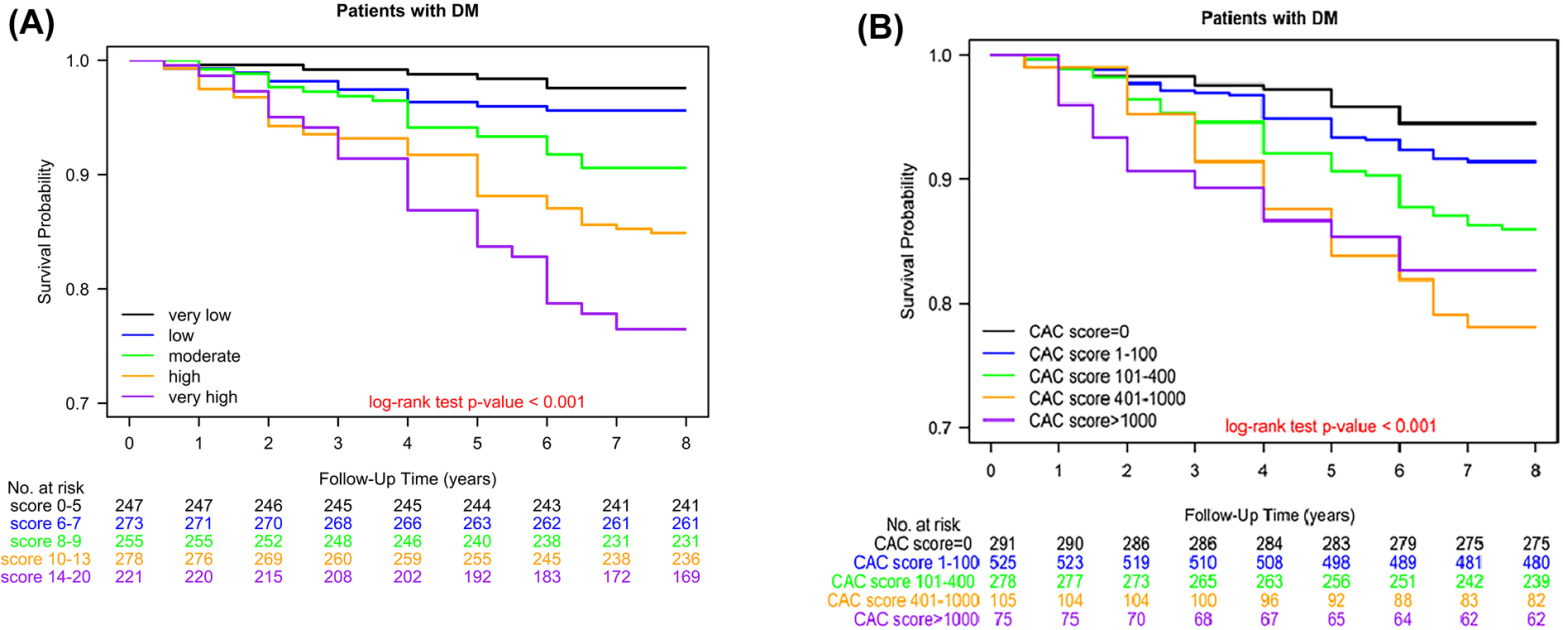
Kaplan–Meier overall survival curves for Type 2 diabetic patients with atherosclerotic cardiovascular disease risk factors persisting for over 5 years, stratified by **A** Poh-Ai predictive and **B** CAC scoring systems into five groups

Patients were stratified into five distinct groups based on risk scores derived from the Poh-Ai predictive scoring system: very low-risk (score 0–5), low risk (score = 6–7), moderate-risk (score = 8–9), high-risk (score = 10–13), and very high-risk groups (score = 14–20). These risk groups exhibited significant disparities in 5-year overall survival rates (very low-risk: 98.8%, low-risk: 97.1%, moderate risk: 94.5%, high-risk: 87.2%, and very high-risk: 83.4%). Statistical analysis confirmed the significance of these differences (log-rank test P < 0.001), as depicted in Fig. 2A. Moreover, the 5-year cumulative overall survival varied significantly among the five groups defined by CAC scores (score 0: 96.4%, score 1–100: 94.9%, score 101–400: 90.7%, score 401–1000: 84.3%, and score > 1000: 86.6%). This distinction was also statistically significant (log-rank test P < 0.001), as shown in Fig. 2B.

### Area under the curve of Poh-Ai and CAC scores for cardiac mortality and revascularization

The areas under the ROC curves, specifically for cardiac mortality, were 0.63 for CAC scores and 0.72 for Poh-Ai scores (as illustrated in Supplemental Fig. 1A), with the Delong test confirming their statistical significance (Delong test < 0.001). Likewise, when assessing revascularization, the areas under the ROC curves were 0.61 for CAC scores and 0.72 for Poh-Ai scores (as presented in Supplemental Fig. 1B). This distinction was also verified as statistically significant by the Delong test (Delong test < 0.001).

### Validating the Poh-Ai prediction model

We recognize the need for validation in a different population, but due to limitations, we used bootstrap sampling as an alternative [17]. This method enhances model credibility by drawing repeated samples with replacement from the original data [18]. Bootstrap resampling allows for the derivation of performance distributions and evaluation of model stability [17]. We utilized the DeLong test to compare diagnostic test performance and conducted Kaplan–Meier analysis for mortality evaluation, with statistical significance set at P < 0.05. Our results show no significant difference in ROC curve areas (Supplemental Fig. 2, P = 0.980), indicating the robustness of our scoring system despite validation constraints.

## Discussion

The CAC score serves as a valuable clinical asset, primarily excelling in the early detection of coronary artery disease (CAD) [1–3]. Its utility allows for prompt intervention and personalized risk evaluation, thus enabling healthcare providers to tailor treatment strategies for individual patients [4–7]. Although numerous studies have confirmed its efficacy in predicting coronary events and the severity of CAD [19, 20], it is essential to acknowledge certain limitations. Specifically, the CAC scoring system's primary focus remains largely centered on evaluating CAD risk, which restricts its suitability for forecasting a wider range of health conditions. To effectively predict all-cause mortality, especially in populations with intricate risk factors such as T2DM and ASCVD risk factors extending over five years, a more holistic approach becomes imperative. Our approach should encompass a wider array of variables, including comorbidities, medication profiles, and lifestyle components (Table 1). An enhanced predictive tool that amalgamates the strengths of CAC scores with these additional variables is crucial for improving patient care and outcomes. By discerning individuals at the highest risk of mortality, timely interventions can be implemented to enhance patient prognoses [14]. This underscores the vital role of the newly developed Poh-Ai predictive tool in augmenting the capacity to identify and mitigate the risk of all-cause mortality in complex patient populations.

The CAC score, derived from coronary artery calcium scanning, plays a pivotal role in assessing calcium deposits within coronary arteries, enabling early detection of heart disease and personalized risk management. Notably, several studies have emphasized the potential of CAC scores in predicting all-cause mortality and improving risk assessment methodologies [21–23]. Our investigation unveiled a significant association between CAC scores and all-cause mortality, with mortality rates rising as cumulative risk scores increased. However, it's essential to acknowledge that the predictive accuracy of CAC scores diminishes when the score exceeds 1000, leading to less reliable predictions of all-cause mortality (Table 4 and Fig. 1). Notably, patients with CAC scores above 1000 demonstrated superior overall survival compared to those with scores ranging from 401 to 1000, indicating potential limitations in predicting all-cause mortality by CAC scores alone (Poh-Ai predictive scores, complemented by additional factors [see Table 2], provide enhanced accuracy in predicting all-cause mortality among individuals with T2DM and ASCVD risk factors persisting for over 5 years) (Fig. 2B).

The mortality risk in individuals with T2DM and ASCVD over a period of more than 5 years is a concerning issue [9–13]. Several factors contribute to the higher mortality rates observed in this specific patient population [10]. Disease progression is a significant contributor, as both T2DM and ASCVD can worsen over time, leading to increased complications and mortality [9–13]. Chronic inflammation, commonly associated with both conditions, can further damage the cardiovascular system and exacerbate the risk [24, 25]. Moreover, individuals with T2DM and ASCVD risk factors often have comorbidities such as hypertension, obesity, and dyslipidemia, which collectively elevate their mortality risk [26, 27]. Additionally, the lack of symptomatic manifestations in asymptomatic patients may delay interventions, allowing the conditions to progress [28]. Lastly, age plays a role, with older T2DM adults naturally having a higher risk of mortality [29]. Once individuals at high risk are identified, preventive measures can be implemented. These may include more frequent medical check-ups and monitoring, adjustments to medications, personalized treatment plans, and a stronger focus on patient engagement [10]. Early interventions are vital in mitigating risks associated with T2DM and ASCVD [6, 7]. Providing education and emotional support further encourages patients to adhere to their treatment and lifestyle plans. Addressing the elevated mortality risk in asymptomatic T2DM patients with ASCVD for over 5 years requires a comprehensive approach, including risk assessment, preventive strategies, and early interventions. The Poh-Ai predictive models play a pivotal role in identifying high-risk individuals, enabling tailored care to reduce mortality effectively (Tables 3 and 4, and Fig. 1).

**Table 3:** Evaluation of all-cause mortality using the Poh-Ai predictive scoring system in Type 2 diabetic patients with atherosclerotic cardiovascular disease risk factors persisting for over 5 years

Poh-Ai predictive scoring system	Number of surviving patients	Number of deaths	Mortality incidence %
0	11	0	0.0
1	7	0	0.0
2	21	0	0.0
3	29	2	6.5
4	79	1	1.3
5	94	3	3.1
6	118	7	5.6
7	143	5	3.4
8	127	9	6.6
9	104	15	12.6
10	88	13	12.9
11	47	8	14.6
12	50	13	20.6
13	51	8	13.6
14	68	14	17.1
15	37	8	17.8
16	43	16	27.1
17	7	2	22.2
18	14	11	44.0
20	0	1	100.0

**Table 4:** Evaluation of all-cause mortality using the Poh-Ai predictive scoring system and CAC scores

Poh-Ai predictive scoring system	Group	Type 2 diabetes patients, N (%)	Mortality among Type 2 diabetes patients, N (%)
Score			
0–5	Very Low risk	247 (19%)	6 (2%)
6–7	Low risk	273 (21%)	12 (4%)
8–9	Moderate risk	255 (20%)	24 (9%)
10–13	High Risk	278 (22%)	42 (15%)
14–20	Very High risk	221 (17%)	52 (24%)

CAC Scoring System	Group	Type 2 diabetes patients, N (%)	Mortality among Type 2 diabetes patients, N (%)
Score			
0	Very Low risk	291 (23%)	16 (5.5%)
1–100	Low risk	525 (42%)	45 (8.6%)
101–400	Moderate risk	278 (22%)	39 (14.0%)
401–1000	High Risk	105 (8%)	23 (21.9%)
CAC > 1000	Very High risk	75 (6%)	13 (17.3%)

*CAC*: Coronary Artery Calcium; *N*: Numbers

The need for a predictive scoring system for all-cause mortality in asymptomatic individuals with T2DM and ASCVD risk factors lasting over five years is driven by several critical factors. Firstly, despite the availability and widespread use of tools like the CAC score, these tools primarily focus on cardiovascular outcomes, such as coronary events [1–3], and may not provide a comprehensive assessment of the overall risk of mortality. Secondly, individuals with T2DM and long-standing ASCVD risk factors face a unique set of health challenges that require tailored risk assessment and management strategies [9–13]. The impact of multiple clinical and demographic variables on mortality risk necessitates a more holistic approach to prediction (Fig. 1) [26, 27]. As such, there is a clear gap in risk assessment tools that can accurately predict all-cause mortality in this specific population. A predictive scoring system like Poh-Ai addresses this gap by considering a wide range of risk factors and providing a patient-centered approach (Table 1). By distinguishing itself from CAC scores and other traditional risk assessment tools, Poh-Ai predictive scores aim to better identify high-risk individuals and facilitate timely interventions (Fig. 1 and Supplemental Fig. 1), ultimately contributing to a reduction in mortality for asymptomatic T2DM patients with ASCVD risk factors lasting over five years.

In our analysis, we embarked on a critical endeavor to address the pressing demand for a predictive scoring system that can effectively and accurately evaluate the risk of all-cause mortality in asymptomatic individuals afflicted with T2DM and enduring ASCVD risk factors, spanning a duration of over five years. While conventional instruments like the CAC score have commendably contributed to the assessment of cardiovascular outcomes, they may not offer a holistic evaluation of the overall mortality risk. This lacuna in risk assessment is particularly paramount for individuals grappling with the distinctive health challenges posed by prolonged T2DM and enduring ASCVD risk factors. Our analysis ushered in the innovative Poh-Ai Predictive Scoring System, which takes into account an extensive array of clinical and demographic variables for prognosticating all-cause mortality (Table 1). Our findings unequivocally establish the efficacy of the Poh-Ai scoring system, not only in predicting all-cause mortality but also in assessing cardiac mortality and coronary revascularization (see Fig. 1 and Supplemental Fig. 1, Delong test p-value < 0.001). By amalgamating critical risk factors, we have established a direct correlation between cumulative risk scores and mortality rates, providing a precise method for classifying individuals into distinct risk categories (refer to Table 3). This stratification has the potential to exert a substantial influence on clinical decision-making and interventions, ultimately leading to enhanced patient outcomes. In stark contrast, we also conducted an evaluation of the utility of the CAC score, derived from coronary artery calcium scanning, in predicting all-cause mortality. Although it has exhibited promise in the domain of cardiovascular risk assessment, its accuracy substantially wanes when the CAC score exceeds 1000, resulting in less dependable predictions of all-cause mortality (see Table 4 and Fig. 2). The amassed data further reinforces the fact that the Poh-Ai scoring system outperforms the CAC score in predicting all-cause mortality, cardiac mortality, and coronary revascularization, conclusively underscoring its superior prognostic value (Fig. 1 and Supplemental Fig. 1). Crucially, our results serve to affirm that the Poh-Ai scoring system offers heightened precision in forecasting all-cause mortality among individuals grappling with T2DM and enduring ASCVD risk factors spanning over five years, surpassing the predictive capacity of CAC scores. This underscores the indispensable role of Poh-Ai scores in identifying high-risk patients who stand to benefit from timely interventions, and accentuates the compelling need for a comprehensive predictive model that transcends the confines of traditional risk assessment tools.

Our main objective is to compare and contrast with CAC scores, which are currently based on computed tomography (CT)-based technology, supplemented by additional clinical factors to improve the prediction of all-cause mortality risk. This is the primary focus of our study. The mentioned scores, such as the United Kingdom Prospective Diabetes Study (UKPDS) risk engine [30], SCORE2-Diabetes Working Group [31], European Society of Cardiology (ESC) [32], Australian Type 2 Diabetes Risk Assessment Tool (AUSDRISK) [33], German Diabetes Risk Score [34], Framingham score [35], ASCVD Risk Score Plus [36], QRISK [37], Reynolds [38], and Multi-Ethnic Study of Atherosclerosis (MESA) Risk Core [39], do not utilize CT-based CAC scores. Moreover, most of these scores primarily predict cardiovascular risk, which differs from our primary goal. Comparing our scoring system with all of these would be less relevant and not aligned with the main purpose of our study.

Due to our focus on predicting all-cause mortality rather than heart-related risks like heart failure, we did not include NT-proBNP and/or hs-Troponin concentrations or echocardiogram data [40–44]. Instead, we integrated traditional factors with CAC scores, as they are simpler and more readily available in clinical practice. The variables included CAC scores, age, gender, BMI, waistline measurements, smoking status, and various laboratory parameters. Our study demonstrated that the Poh-Ai Predictive Scoring System outperformed CAC scores alone in predicting mortality and cardiovascular events among T2DM patients with extended atherosclerotic cardiovascular disease risk factors. While additional factors may enhance prediction accuracy, they were not considered due to data unavailability and our focus on all-cause mortality. The Poh-Ai system offers an effective tool for risk assessment in this population, but further investigation into additional variables is warranted.

There is indeed evidence that using a set of relevant clinical and laboratory variables increases the discriminatory power for the risk of outcomes. Our study aligns with this phenomenon, as we added variables encompassing demographic attributes, clinical parameters, comorbidities, and medication usage to the CAC scores, resulting in the development of the Poh-Ai Predictive Scoring System. This system excels in forecasting mortality and cardiovascular events in individuals with Type 2 Diabetes Mellitus and extended ASCVD risk factors. While our study population, consisting of individuals aged 40–80 years with Type 2 Diabetes Mellitus and at least 5 years of ASCVD risk factors, differs from that of the MESA study [39], which included individuals free of clinical heart disease at baseline and followed for 10 years, the integration of traditional factors into CAC scores substantially enhances prediction accuracy. Although the MESA study specifically focused on the accuracy of coronary heart disease (CHD) risk prediction, our study emphasizes the enhancement of prediction accuracy for mortality and cardiovascular events. Thus, while our study and the MESA study differ in population characteristics and outcome measures, both underscore the importance of integrating traditional risk factors into CAC scores to improve risk prediction accuracy [39].

This study has several limitations that should be considered. First, the sample size, while adequate for the purposes of this study, may not capture the full spectrum of patient diversity. The findings are derived from a select group of individuals and may not be entirely generalizable to broader demographics. Second, the retrospective nature of the study introduces inherent biases and limitations associated with this approach. The reliance on preexisting data makes the study susceptible to selection bias and data quality issues. Third, the data used in this analysis was drawn from a population of Asian T2DM patients, which may introduce biases associated with ethnicity. Consequently, the results might not entirely mirror the broader clinical landscape and patient diversity that encompasses different racial groups. Fourth, the choice of variables included in the analysis and the exclusion of others may impact the results. The predictive model's accuracy and reliability are dependent on the variables selected, potentially leaving out other relevant factors. Fifth, there may be unmeasured confounding factors that were not considered in the analysis, which could impact the results. Sixth, temporal changes in cardiovascular management and treatment approaches over the years could potentially impact the outcomes, given the data covers a specific timeframe. Although medication use and the continuous monitoring of all enrolled patients in the study have been maintained, it is important to acknowledge that medication adherence and lifestyle modifications may vary on an individual basis. Seventh, the analysis relies on specific statistical assumptions, and the model's predictive performance is contingent on the validity of these assumptions. Deviations from these assumptions could affect the model’s reliability. Despite these limitations, the study provides valuable insights into improving risk assessment for a specific patient population and underscores the potential of the Poh-Ai scoring system. Future research should address these limitations, explore external validation, and further refine predictive models to enhance the management and outcomes of T2DM patients with prolonged ASCVD risk factors.

## Conclusions

The Poh-Ai Predictive Scoring System demonstrates its superiority in forecasting mortality and cardiovascular events among individuals with Type 2 Diabetes Mellitus and extended ASCVD risk factors. These results underscore the potential for early interventions and enhanced patient outcomes.

### Supplementary Information

Supplementary Material 1: Figure 1. ROC Curves: Areas Under the Curve for Poh-Ai Predictive and CAC Scores in Relation to (A) Cardiac Mortality and (B) Coronary Revascularization. Figure 2. Comparison of Receiver Operating Characteristic (ROC) Curve Areas for Validation of Scoring System in Cancer Patients.

## Data Availability

The datasets supporting the study conclusions are included within this manuscript and its additional files.

## Abbreviations

T2DM: Type 2 diabetes
ASCVD: Atherosclerotic cardiovascular disease
CAC: Coronary artery calcium
AHR: Adjusted hazard ratios
HR: Hazard ratios
BMI: Body mass index
LDL: Low-density lipoprotein
HDL: High-density lipoprotein
HbA1c: Glycated hemoglobin
AIC: Akaike information criterion
ROC: Receiver operating characteristic
AUC: Area under the curve
CAD: Coronary artery disease
