# Supplementary material for: Assessing mortality risk in Type 2 Diabetes patients with prolonged ASCVD risk factors: the inclusive Poh-Ai predictive scoring system with CAC Score integration
Source: Diabetol Metab Syndr. 2024 May 19;16:104. doi: 10.1186/s13098-024-01341-9 (PMC11103845; doi:10.1186/s13098-024-01341-9)
Supplement: Supplementary file 1 — Supplementary Material 1: Figure 1. ROC Curves: Areas Under the Curve for Poh-Ai Predictive and CAC Scores in Relation to (A) Cardiac Mortality and (B) Coronary Revascularization. Figure 2. Comparison of Receiver Operating Characteristic (ROC) Curve Areas for Validation of Scoring System in Cancer Patients. [file 13098_2024_1341_MOESM1_ESM.docx]

**Supplemental Figure 1. ROC Curves: Areas Under the Curve for Poh-Ai Predictive and CAC Scores in Relation to (A) Cardiac Mortality and (B) Coronary Revascularization.**

1. **(B)**


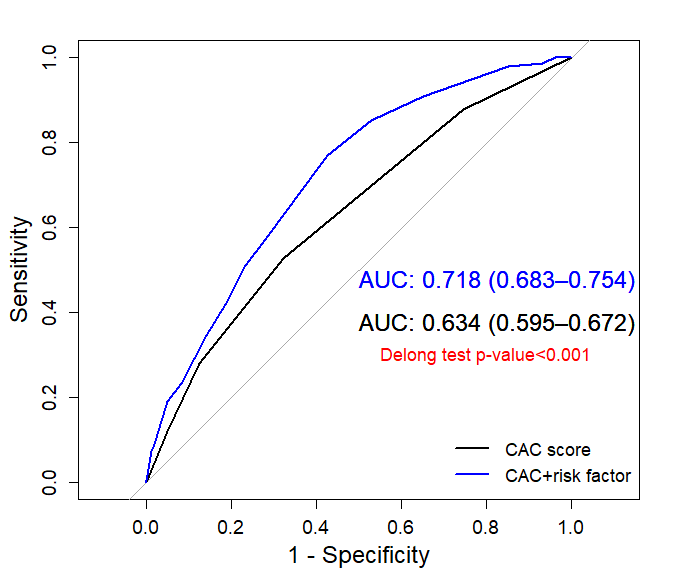

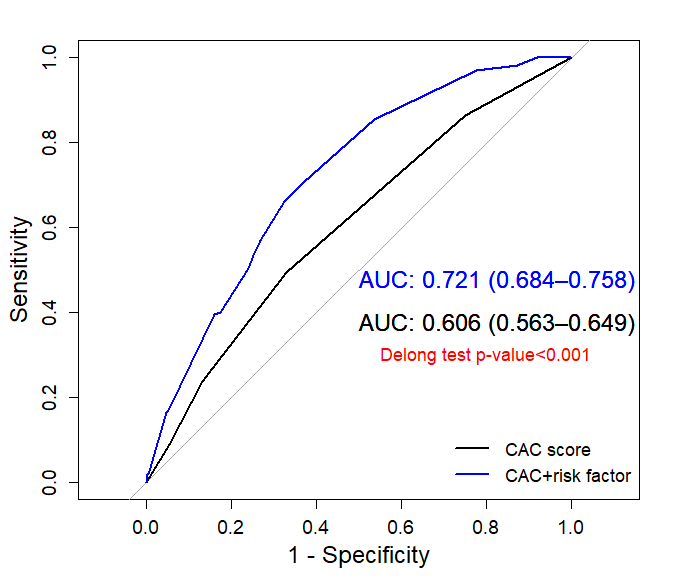


**Supplemental Figure 2. Comparison of Receiver Operating Characteristic (ROC) Curve Areas for Validation of Scoring System in Cancer Patients
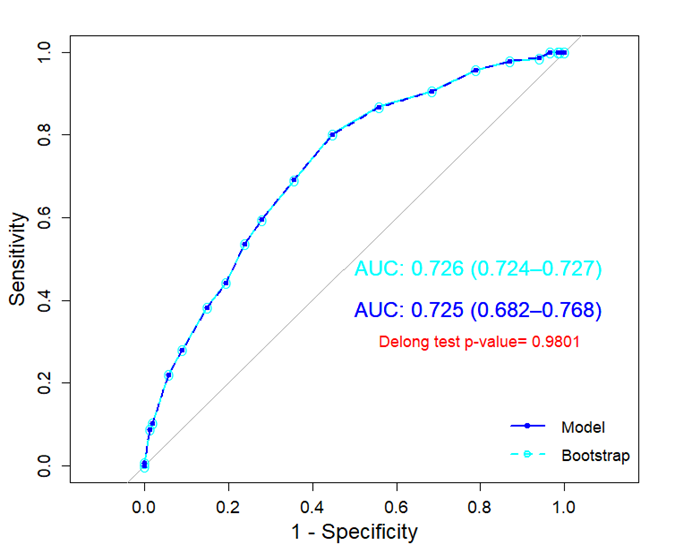
**
